# Hidden blood loss and its possible risk factors in minimally invasive transforaminal lumbar interbody fusion

**DOI:** 10.1186/s13018-020-01971-5

**Published:** 2020-09-29

**Authors:** Yuanxing Zhou, Xin Fu, Ming Yang, Song Ke, Bo Wang, Zhonghai Li

**Affiliations:** 1 Department of Orthopaedics, First Affiliated Hospital of Dalian Medical University, Dalian, People’s Republic of China; 2 Key Laboratory of Molecular Mechanism for Repair and Remodeling of Orthopaedic Diseases, Dalian, Liaoning Province People’s Republic of China; 3 Department of Neurosurgery, First Affiliated Hospital of Dalian Medical University, Dalian, People’s Republic of China

**Keywords:** Hidden blood loss (HBL), Risk factors, Minimally invasive transforaminal lumbar interbody fusion (MIS-TLIF), Multiple regression analysis, Complication

## Abstract

**Background:**

With respect to spinal surgeries, elucidating absolute and relative amount of hidden blood loss (HBL) is of great importance in order to avoid aforementioned potential complications. To evaluate HBL and its possible risk factors among patients undergoing minimally invasive transforaminal lumbar interbody fusion (MIS-TLIF) for lumbar degenerative diseases.

**Methods:**

Between June 2018 and March 2019, 137 consecutive patients with lumbar degenerative disease, who underwent operation with MIS-TLIF technique, were enrolled in this study. The patient’s demographic characteristics and blood loss-related parameters were collected, respectively. The Pearson or Spearman correlation analysis was used to investigate an association between patient’s characteristics and HBL. Multivariate linear regression analysis was used to confirm independent risk factors of HBL.

**Results:**

A total of 137 patients (86 males and 51 females, age range 19–78 years) were reviewed in our hospital. A substantial amount of HBL (488.4 ± 294.0 ml, 52.5% of TBL) occurred after MIS-TLIF. Multivariate linear regression showed that the age, muscle thickness, the Patients’ Society of Anesthesiologists (ASA) classification, patient’s blood volume (PBV), total blood loss (TBL), postoperative (i.e., day 2 or 3) hematocrit (Hct), Hct loss, and fibrinogen level were independent risk factors for HBL (*P*1 = 0.000, *P*2 = 0.002, *P*3 = 0.006, *P*4 = 0.002, *P*5 = 0.003, *P*6 = 0.048, *P*7 = 0.004, *P*8 = 0.000).

**Conclusion:**

A large amount of HBL was incurred in patients undergoing MIS-TLIF. More importantly, the age, muscle thickness, ASA classification, PBV, TBL, postoperative Hct, Hct loss, and fibrinogen level were independent risk factors for HBL in MIS-TLIF. HBL and its risk factors should be paid more attention to during the perioperative period.

## Introduction

Hidden blood loss (HBL) is not usually recognized by general assessment because of its invisibility, while an association is found between increased blood loss and perioperative complications [1]. HBL can exacerbate postoperative hemoglobin drop, leading to increased transfusion requirement: if not properly managed, it may induce delayed wound healing, increased risk of infection, and prolonged postoperative rehabilitation. Since Sehat et al. [2] reported that HBL following total hip replacement was 49% of the total blood loss, surgeons became aware that HBL plays an important role in orthopedic procedures. However, HBL is still not well known or used in the setting of spine surgery. With respect to spinal surgeries, elucidating absolute and relative amount of HBL is of great importance in order to avoid aforementioned potential complications.

Minimally invasive transforaminal lumbar interbody fusion (MIS-TLIF) has gained popularity as an alternative for the treatment of lumbar degenerative diseases thanks to several superiorities, such as minimized surgical trauma, accelerated postoperative rehabilitation, less postoperative complications, and reduced intraoperative bleeding [3–6]. In clinical practice, however, there still exist a large number of patients suffering from anemia or related disorders after this minimally invasive surgery. Moreover, the degree of postoperative anemia turns out to be not in accordance with the amount of perioperative blood loss. Sehat et al. [2] proposed the concept of HBL in 2000, which might be in association with negative postoperative outcomes [7, 8]. According to published studies, the HBL in lumbar fusion surgery ranged from 227 to 600 ml, but most surgeons might ignore it [9–11]. A previous study showed that compared with open transforaminal lumbar interbody fusion (O-TLIF), HBL in patients undergoing MIS-TLIF was seriously underestimated and accounted for a larger percentage of total blood loss (TBL) even though TBL after MIS-TLIF was much less [12]. To the best of our knowledge, there was no research that analyzed the risk factors of HBL in MIS-TLIF. This study could be the first one to investigate this field. Therefore, we retrospectively reviewed medical data of patients who underwent MIS-TLIF in our department in an attempt to evaluate HBL and identify its risk factors.

## Materials and methods

### Patient population

This was a retrospective clinical study. From June 2018 to March 2019, 137 patients having lumbar degenerative disease at our institution (First Affiliated Hospital of Dalian Medical University) were included in this study. Information gathered included demographic details, etiology, diagnosis, radiological, and laboratory investigations. Pre-, intra-, and postoperative findings were recorded as well. All patients aged 18 years or older who had lumbar degenerative diseases (lumbar canal stenosis, spondylolisthesis, and lumbar disk herniation) treated by MIS-TLIF by only one experienced surgeon were included. Exclusion criteria were patients (1) age less than 18 years; (2) with lumbar infections and tumors; (3) with previous lumbar surgery; (4) unexpectedly suffered dural rupture during surgery; (5) with acute lumbar fracture; (6) combined with blood-related diseases, coagulopathy, and severe anemia; (7) with antiplatelet drugs or anticoagulants; (8) with autologous and allogeneic blood transfusion; and (9) with intraoperative blood loss greater than 1.5 l [13].

### Data extraction

**Fig. 1:**
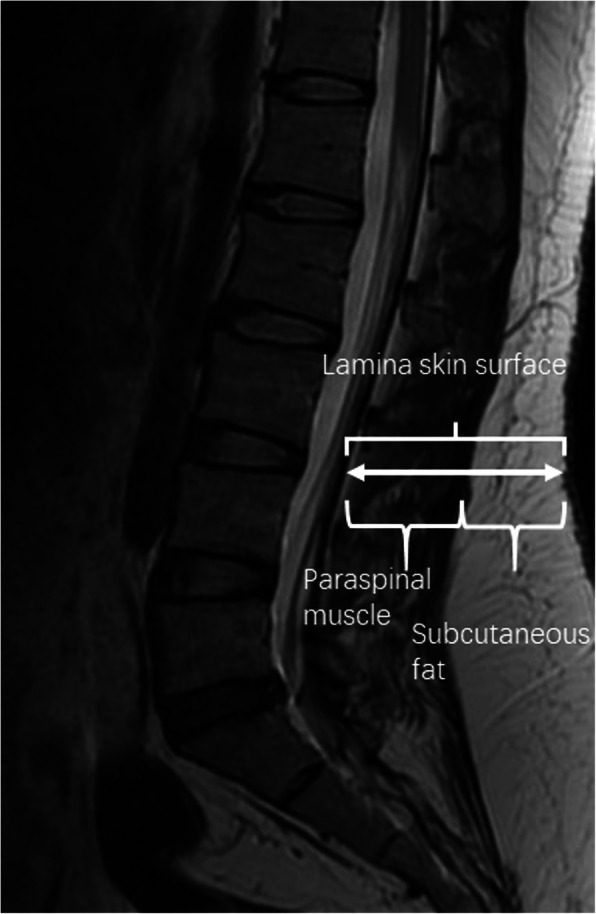
Diagram of the method used to measure the paraspinal muscle, subcutaneous fat, and lamina at the skin surface at the level of L4 using sagittal views was determined on T2-weighted MRI

Patient data were collected from the electronic medical records system of our institution. Demographic characteristics such as sex, age, weight, height, body mass index (BMI), hypertension (i.e., blood pressure ≥ 140/90 mmHg), diabetes mellitus (i.e., fasting blood-glucose ≥ 6.1 mmol/l), smoking, drinking, surgical duration, muscle thickness, subcutaneous fat thickness, hospital stay, the Patients’ Society of Anesthesiologists (ASA) classification, and level fused were assessed and recorded. Blood loss-related data such as intraoperative blood loss, preoperative hematocrit (Hct), preoperative hemoglobin (Hb), postoperative (i.e., day 2 or 3) Hct, postoperative (i.e., day 2 or 3) Hb, prothrombin time (PT), activated partial thromboplastin time (APTT), thrombin time (TT), fibrinogen, and platelet (PLT) were extracted, respectively. The Hb was aimed to define anemia (i.e., < 120 g/l for females and < 130 g/l for males) [14]. Preoperative magnetic resonance imaging (MRI) was used to determine the distance of the lamina from the skin surface, thickness of the paraspinal muscles, and thickness of the subcutaneous fat. These measurements were all performed at the level of L4, using sagittal views (Fig. 1). To prevent interobserver variability, measurements were performed three times by the same observer who was blinded to the operative details.

### Calculation of hidden blood loss

Firstly, patient’s blood volume (PBV) should be calculated according to the formula of Nadler et al. [15]: PBV = k1 × height(m)^3^ + k2 × weight (kg) + k3 (for male: k1 = 0.3669, k2 = 0.03219, and k3 = 0.6041; for female: k1 = 0.3561, k2 = 0.03308, and k3 = 0.1833). Secondly, according to the Gross formula [16], total blood loss (TBL) was calculated by multiplying PBV by changes of Hct: TBL = PBV (Hct_pre_ − Hct_post_)/Hct_ave_, where Hct_pre_ is the preoperative Hct, Hct_post_ is the second or third postoperative Hct, and Hct_ave_ is the average of Hct_pre_ and the Hct_post_. Consequently, we calculated the hidden blood loss according to the formula of Sehat et al. [17]: HBL = TBL − visible blood loss (VBL). Since all the cases did not have postoperative wound drainage, intraoperative blood loss was equal to VBL, which was calculated as the sum of blood in suction containers and soaked gauzes and sponges.

### Statistical analysis

Data analysis was performed with the SPSS 22.0 software (International Business Machines Corporation, Armonk, NY). Student’s *t* test was used to compare differences between pre- and postoperative Hct values and Hb levels. The chi-squared test was taken to compare pre- and postoperative anemia. Pearson’s correlation (used for the normal data), Spearman’s correlation analysis (used for the non-normal data), and multivariate linear regression analysis were established to identify risk factors associated with the HBL, such as gender, age, BMI, hypertension, diabetes mellitus, smoking, drinking, surgical duration, muscle thickness, subcutaneous fat thickness, hospital stay, ASA classification PBV, VBL, TBL, preoperative Hct, preoperative Hb, postoperative Hct, postoperative Hb, PT, APTT, TT, PLT, and level fused. *P* < 0.05 was considered statistically significant.

## Results

A total of 137 patients (86 males and 51 females, age range 19–78 years) were retrospectively reviewed in this study. The demographic information is summarized in Table 1. The mean muscle thickness was 38.8 ± 6.6 mm, while the mean subcutaneous fat thickness was 27.0 ± 5.1 mm. The mean preoperative Hct and Hb were 38.8 ± 4.5 and 121.1 ± 16.5 g/l. The mean postoperative Hct and Hb were 32.8 ± 4.5 and 105.9 ± 16.7 g/l. The mean PBV was 5.0 ± 0.7 l. The mean HBL was 488.4 ± 294.0 ml, 52.5% of TBL. The mean VBL was 284.2 ± 108.4 ml. The mean TBL was 772.5 ± 328.8 ml. Hct loss was 5.5 ± 2.1 and Hb loss was 15.2 ± 7.8 g/l. There were significant differences between pre- and postoperative Hct (*P* < 0.001) and Hb (*P* < 0.001), and 42 patients developed anemia after surgery (*P* < 0.001, Table 2).

**Table 1:** Patients demographics

Parameters	Statistics
Total patients (*n*)	137
Sex (*n*)	
Male	86
Female	51
Age, yr	49.6 ± 9.5
BMI, kg/m^2^	26.0 ± 3.9
Hypertension (*n*)	35
Diabetes mellitus (*n*)	31
Smoking (*n*)	50
Drinking (*n*)	28
Surgical duration, min	127.7 ± 52.7
Muscle thickness, mm	38.8 ± 6.6
Subcutaneous fat thickness, mm	27.0 ± 5.1
Hospital stay, d	11.4 ± 2.7
ASA classification (*n*)	
I	47
II	56
III	27
IV	7
PBV, L	5.0 ± 0.7
VBL, ml	284.2 ± 108.4
HBL, ml	488.4 ± 294.0
TBL, ml	772.5 ± 328.8
HBL/TBL (%)	52.5 ± 18.6
Preoperative Hct	38.8 ± 4.5
Preoperative Hb, g/l	121.1 ± 16.5
Postoperative Hct	32.8 ± 4.5
Postoperative Hb, g/l	105.9 ± 16.7
Hct loss	5.5 ± 2.1
Hb loss, g/l	15.2 ± 7.8
PT, s	11.7 ± 1.0
APTT, s	28.6 ± 6.3
TT, s	13.6 ± 1.3
Fibrinogen, g/l	3.3 ± 0.8
PLT, 10^9^/l	255.1 ± 68.2
Level fused (*n*)	
One level	91
Two levels	41
Three levels	6

*BMI* Body mass index, *ASA* American Society of Anesthesiologists, *PBV* Patient’s blood volume, *VBL* Visible blood loss, *HBL* Hidden blood loss, *TBL* Total blood loss, *Hct* Hematocrit, *Hb* Hemoglobin, *PT* Prothrombin time, *APTT* Activated partial thromboplastin time, *TT* Thrombin time, *PLT* Platelet, *yr* Year, *d* Day

**Table 2:** Changes in Hct, Hb, and anemia level following minimally invasive transforaminal lumbar interbody fusion (MIS-TLIF)

	Preoperative (***n*** = 137)	Postoperative (***n*** = 137)	Statistical significance
Hct, %	38.2 ± 4.5	32.8 ± 4.5	*P* < 0.001
Hb, g/l	121.1 ± 16.5	105.9 ± 16.7	*P* < 0.001
Anemia	76	118	*P* < 0.001

*Hct* Hematocrit, *Hb* Hemoglobin

The Pearson or Spearman correlation analysis for HBL found the following parameters with a *P* < 0.05 (Table 3): age (*P* = 0.000), surgical duration (*P* = 0.000), muscle thickness (*P* = 0.000), subcutaneous fat thickness (*P* = 0.000), ASA classification (*P* = 0.000), PBV (*P* = 0.000), TBL (*P* = 0.000), postoperative Hct (*P* = 0.000), postoperative Hb (*P* = 0.000), Hct loss (*P* = 0.000), Hb loss (*P* = 0.000), APTT (*P* = 0.000), fibrinogen level (*P* = 0.000), and level fused (*P* = 0.000). Multivariate linear regression showed that the age, muscle thickness, ASA classification, PBV, TBL, postoperative Hct, Hct loss, and fibrinogen level were independent risk factors for HBL (*P*1 = 0.000, *P*2 = 0.002, *P*3 = 0.006, *P*4 = 0.002, *P*5 = 0.003, *P*6 = 0.048, *P*7 = 0.004, *P*8 = 0.000, Table 4).

**Table 3:** Results of the Pearson or Spearman correlation analysis for hidden blood loss

Parameters	Sig (2-tailed)	***P***
Gender	0.052	0.550
Age	0.689	**0.000**
BMI	− 0.104	0.229
Hypertension	0.054	0.533
Diabetes mellitus	0.088	0.308
Smoking	− 0.128	0.136
Drinking	0.109	0.203
Surgical duration	0.611	**0.000**
Muscle thickness	0.794	**0.000**
Subcutaneous fat thickness	0.466	**0.000**
Length of stay	− 0.071	0.409
ASA classification	0.790	**0.000**
PBV	0.333	**0.000**
VBL	0.152	0.077
TBL	0.945	**0.000**
Preoperative Hct	− 0.009	0.917
Preoperative Hb	− 0.062	0.470
Postoperative Hct	− 0.398	**0.000**
Postoperative Hb	− 0.327	**0.000**
Hct loss	0.832	**0.000**
Hb loss	0.570	**0.000**
PT	− 0.130	0.131
APTT	− 0.331	**0.000**
TT	0.025	0.770
Fibrinogen	− 0.873	**0.000**
PLT	0.049	0.568
Level fused	0.567	**0.000**

Values in bold indicate statistical significance

*BMI* Body mass index, *ASA* American Society of Anesthesiologists, *PBV* Patient’s blood volume, *VBL* Visible blood loss, *TBL* Total blood loss, *Hct* Hematocrit, *Hb* Hemoglobin, *PT* Prothrombin time, *APTT* Activated partial thromboplastin time, *TT* Thrombin time, *PLT* Platelet

**Table 4:** Results of multivariate linear regression for hidden blood loss

Coefficients^**a**^	Unstandardized		Standardized		
	***β***	SE	***β***	***t***	***P***
Constant	− 145.312	135.160		− 1.075	0.284
Age	3.279	0.907	0.106	3.616	**0.000**
Surgical duration	− 0.154	0.209	− 0.028	− 0.739	0.461
Muscle thickness	5.683	1.754	0.127	3.241	**0.002**
Subcutaneous fat thickness	− 1.487	1.564	− 0.026	− 0.951	0.344
ASA classification	32.196	11.528	0.101	2.793	**0.006**
PBV	58.611	18.259	0.139	3.210	**0.002**
TBL	0.279	0.091	0.313	3.071	**0.003**
Postoperative Hct	− 5.062	2.531	− 0.077	− 2.001	**0.048**
Postoperative Hb	0.150	0.404	0.008	0.370	0.712
Hct loss	39.861	13.592	0.283	2.933	**0.004**
Hb loss	0.860	1.060	0.023	0.811	0.419
APTT	− 1.867	1.579	− 0.040	− 1.183	0.239
Fibrinogen	− 72.723	15.446	− 0.199	− 4.708	**0.000**
Level fused	− 33.994	18.593	− 0.066	− 1.828	0.070

Values in bold indicate statistical significance

*ASA* American Society of Anesthesiologists, *PBV* patient’s blood volume, *HBL* hidden blood loss, *TBL* total blood loss, *Hct* hematocrit, *Hb* hemoglobin, *APTT* activated partial thromboplastin time

^a^Dependent variable: HBL (ml)

## Discussion

Spinal fusion surgery associated with excessive blood loss has been documented [18–20]. So, the concept of HBL was proposed in 2000 [2]. HBL is now paid attention to and considered as an important proportion of total blood loss, but instead, it remains underestimated by most orthopedic surgeons [17]. Jiang et al. [21] believed that the mean HBL was 337 ml, which was 46.8% of TBL after cervical open-door laminoplasty (EOLP). Ju et al. [10] held that HBL for patients who received anterior lumbar interbody fusion (ALIF) was about 450 ml and averaged 39.2% of TBL. Our result showed that a substantial amount of HBL (488.4 ± 294.0 ml, 52.5% of TBL) frequently occurred after MIS-TLIF, which was quite larger than expected. Nevertheless, the influential factors correlated to the HBL were not confirmed. In our study, we investigated and identified the risk factors of HBL following this surgery by multivariate linear regression analysis. The results proposed that the age, muscle thickness, ASA classification, PBV, TBL, and Hct loss were positive independent risk factors of HBL, while postoperative Hct and fibrinogen level were negatively related to HBL.

ASA classification is reportedly an independent risk factor of HBL in anterior cervical fusion (ACF) surgery [22]. The author held that the HBL of patients with ASA classification III was higher than ASA I and ASA II. What is more, some scholars proposed that higher ASA classification was an independent risk factor for blood transfusion in spine fusion surgery [23, 24]. In our series, we obscured a similar outcome. That is, patients with higher ASA classification seem to take more risk attributing to HBL in MIS-TLIF. As ASA classification is defined, the patients with ASA II to IV usually combine with mild or severe systemic diseases. In other words, these patients’ function of hemodynamics is out of order and they are apt to have less tolerance toward anemia.

PBV was an independent risk factor of HBL in multiple linear regression analysis. In our study, which might relate to the patient’s weight and height, PBV was calculated according to the formula of Nadler et al. [15]. Although BMI is also calculated by weight and height, BMI was not clarified as a risk factor in our study. In addition, our result showed that TBL was another independent risk factor, which may have to do with PBV, because TBL was calculated by multiplying PBV by changes of Hct according to the Gross formula [16]. Based on collected data in our study, the patients with larger TBL were in accordance with higher HBL. Furthermore, postoperative Hct and Hct loss were suggested as independent factors in our series, but not postoperative Hb and Hb loss. Nonetheless, Hct and Hb were both significant differences between pre- and postoperative by Student’s *t* test. Some studies suggested that postoperative fluid dilution should be a vital reason to attribute to more Hct change [25, 26], which might be a possible explanation for different significances between Hct- and Hb-related indexes in multiple linear regression analysis.

Our study found that the fibrinogen level was negatively related to HBL. Fibrinogen refers to blood coagulation factor I, which is the major protein in the process of clotting cascade. Wen et al. [18] indicated that a disproportionate increase in HBL seems to appear for fibrinogen level ≥ 3 versus < 3 fibrinogen level ≥ 2 or < 2 fibrinogen level ≥ 1. That is, the fibrinogen level was a positive influential factor. After careful consideration and analysis, we enable to explain why two studies have contrary outcomes. It is true that patients with a higher fibrinogen level are in accordance with hypercoagulation. In their study, patients had postoperative wound drainage after posterior lumbar fusion (PLF). So, when they calculated HBL, they should minus postoperative drainage. However, bleeding likely coagulated in the lacunae or dead space among the patients with a higher fibrinogen level, decreasing the volume of postoperative drainage. Then, HBL would be calculated larger. On the contrary, the patients in our study were not provided with wound drainage. Thus, all of postoperative hemorrhage could be seen as HBL. Patients with a higher fibrinogen level are liable to form clots and stop bleeding by themselves. Consequently, in our study, the fibrinogen level is a positive influential factor of HBL.

In our study, age has a significant correlation with HBL. A previous study proposed that age was the risk factor of HBL in posterior lumbar fusion (PLF), especially for the age of 60 years or above [18]. One possible explanation is that older patients have a poor compensatory capacity of the cardiovascular system and reduced self-regulatory ability due to angiosclerosis. Another reason might be that bleeding is liable to infiltrating and agglutinating more easily into interstitial spaces, owing to muscle wastage and hypercoagulability in senile patients [27].

Our study firstly considered that muscle thickness was the key factor in predicting HBL in MIS-TLIF, which has not been reported before. We found that muscle thickness was regarded as an index parameter to indicate HBL in lumbar fusion surgery. Surgical techniques may lead to postoperative bleeding on not only bony surfaces and the spinal canal, but also soft tissue dissection including muscle and subcutaneous fat. Subcutaneous fat thickness did not turn out to be a risk factor of HBL in our study, whereas muscle thickness was. The explanation for this is that thicker muscle probably suffers more soft tissue injury which would increase perioperative bleeding. Meanwhile, thicker muscle may be associated with larger penetrable tissue compartments, allowing blood to ooze into the tissue cavity [28].

It has been reported that a large amount of HBL after spine fusion surgery can bring about adverse consequences, such as lengthened hospitalization time, prolonged postoperative rehabilitation, and affected patient satisfaction [29]. The mechanisms of HBL have not been entirely clear. HBL generally ascribes to two pathways: infiltration of bleeding into the tissue compartment or dead cavities and hemolysis. Erskine et al. [17] suggested that 60% of HBL was caused by infiltration of bleeding and 40% by hemolytic reactions, while Sehat et al. [30] believed that the proportion of HBL from extravasation of bleeding and hemolysis was 2:1. In any case, the patient’s Hct and Hb should be checked closely before and after surgery to ensure if the patient has anemia or tendency to anemia. Besides, surgeons also need to give priority to the patient’s age, muscle thickness, ASA classification, PBV, TBL, and fibrinogen level to evaluate that the patient is not at an increased risk of bleeding after surgery.

### Study limitations

Some limitations should be considered in our study. Since this is a descriptive study, it has potential limitations. The number of patients included in this study was relatively small. In addition, whether fluid shift and hemodynamics become stable after 2 to 3 days after spine fusion surgery has not been ascertained. Again, more studies are required to find the accurate time of stability of fluid shift. Finally, we were unable to investigate the influence of racial differences for HBL, because most patients included in our hospital were native residents. Due to these limitations, high-quality observational studies and basic experimental studies are still needed to investigate new risk factors for HBL among patients undergoing MIS-TLIF further in the future.

## Conclusions

Consequently, it concluded that a large amount of HBL was incurred in patients undergoing MIS-TLIF. More importantly, the age, muscle thickness, ASA classification, PBV, TBL, postoperative Hct, Hct loss, and fibrinogen level were independent risk factors for HBL in MIS-TLIF. HBL and its risk factors should be paid more attention to during the perioperative period. Adequate management of the risk factors will help to reduce surgical patients’ morbidity, mortality, and length of stay and save costs for the healthcare institutions.

## Data Availability

All data used and analyzed during this study are available from the corresponding author upon reasonable request.

## Abbreviations

BMI: Body mass index
ASA: American Society of Anesthesiologists
PBV: Patient’s blood volume
VBL: Visible blood loss
HBL: Hidden blood loss
TBL: Total blood loss
Hct: Hematocrit
Hb: Hemoglobin
PT: Prothrombin time
APTT: Activated partial thromboplastin time
TT: Thrombin time
PLT: Platelet
